# Geographical Flushing of the Children's Face: A New Clinical Entity?

**DOI:** 10.1155/2013/802130

**Published:** 2013-05-09

**Authors:** Masatoshi Jinnin, Satoshi Fukushima, Yuji Inoue, Hironobu Ihn

**Affiliations:** Department of Dermatology and Plastic Surgery, Faculty of Life Sciences, Kumamoto University, 1-1-1 Honjo, Kumamoto 860-8556, Japan

## Abstract

A lot of skin diseases can occur on children's faces. We report two children with unique flushing on their faces, sometimes accompanied with headache. Their eruption did not meet various differential diagnoses. Many dermatologists may have seen a similar condition but did not think much about it. Thus, it should be worth regarding such symptom as the benign new clinical entity, which may comfort patients with similar conditions and merit further attention in clinical practice.

## 1. Introduction

A lot of skin diseases can occur on children's faces. Especially, we often see pediatric patients with a “red face” induced by, for example, urticaria, contact dermatitis, atopic dermatitis, or sunburn [1–4]. Recently, we experienced two children with unique flushing on the faces, sometimes accompanied with headache. Because their eruption did not meet various differential diagnoses, we suspect they may be a new clinical entity. 

## 2. Case Report

### 2.1. Case 1

**Figure 1:**
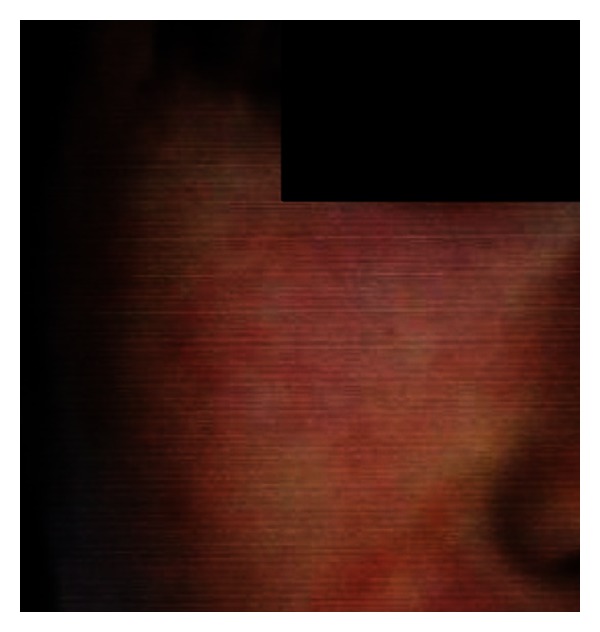
Facial geographical erythemas of case 1.

An 8-year-old Japanese boy visited our hospital, for the treatment of an eruption. One year ago, his parents had noticed multiple reddish macules on his face, when he exercised under the summer sun. Patchy multiple erythemas merged to be geographical (Figure 1), and only the face was involved. The severity of his eruption was proportional to the intensity of exercise, and the rash was sometimes accompanied with sweating and headache. He did not notice pruritus or respiratory distress, and his eruption always regressed spontaneously after about 30 minutes rest.

A clinical diagnosis of solar urticaria was made by an other pediatric clinic, and sunscreen and oral antihistamine drug were tried, but the symptom was not prevented. Then, he was suspected of having photosensitivity (i.e., xeroderma pigmentosum or porphyria) and was referred to our hospital.

We could not find any other abnormalities indicating photosensitivity: minimal response dose by UVA and minimum effective dose by UVB were within normal limits. There was no record of any similar condition in his family history nor did he have an allergy. The patient is currently observed without any treatment, but the symptom did not affect his daily lifes and there have been no other severe problems.

### 2.2. Case 2

**Figure 2:**
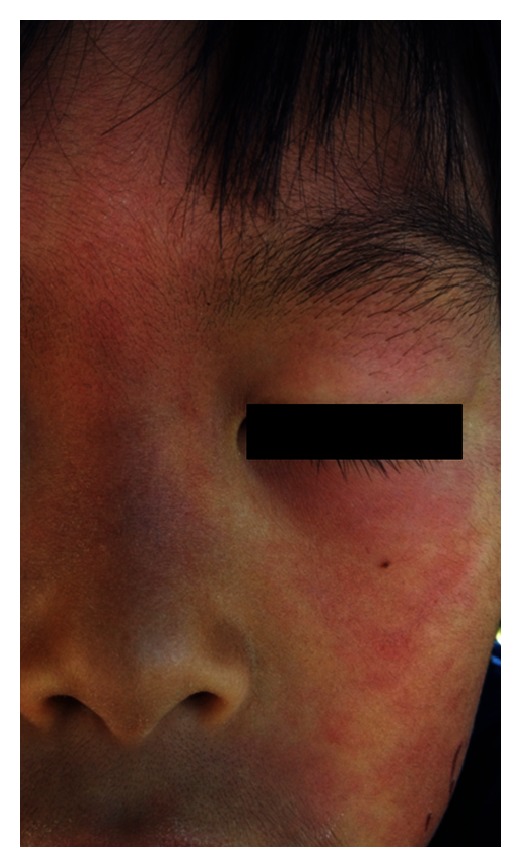
Patchy multiple erythemas on the face of case 2.

An 8-year-old Japanese boy suffered from an eruption during exercise in summer 2 years ago. The eruption was patchy multiple erythemas (Figure 2), sometimes accompanied with sweating and headache. The eruption disappeared without pigmentation or scar after 30 minutes. He was carefully observed without treatment, and the frequency was decreasing gradually.

## 3. Discussion

We considered urticaria, contact dermatitis, photosensitivity (including xeroderma pigmentosum or porphyria), auriculotemporal syndrome (Frey syndrome), and erythema infectiosum as the differential diagnoses of the eruption. Urticaria was denied by the localization on only the face and the lack of pruritus or respiratory distress, contact dermatitis by the spontaneous regression, photosensitivity by the spontaneous regression and the results of photo tests, Frey syndrome by the unrelatedness with eating or thinking of food, and erythema infectiosum by the disease duration of more then 1 year [1–4].

For the Japanese boys, unique geographical distribution on the face and occasional headache were the common factors to our patients. The patients and their parents were anxious about the eruption, because the eruption was very conspicuous on the face. However, during the observation for 1-2 years, no other severe problems have occurred. Thus, it should be worth regarding such a symptom as the benign new clinical entity, which may comfort patients with similar conditions and merit further attention in clinical practice. As far as we searched, we could not find similar cases in previous literatures. Many dermatologists may have seen such a condition but did not think much about it.

To establish the disease concept, accumulation of patient number and additional examination by skin biopsy or photo patch test are needed in the future.
